# Age-dependent influenza infection patterns and subtype circulation in Denmark, in seasons 2015/16 to 2021/22

**DOI:** 10.2807/1560-7917.ES.2024.29.4.2300263

**Published:** 2024-01-25

**Authors:** Hanne-Dorthe Emborg, Amanda Bolt Botnen, Jens Nielsen, Lasse S. Vestergaard, Frederikke Kristensen Lomholt, Charlotte Munkstrup, Karina Lauenborg Møller, Charlotte Kjelsø, Steen Hulthin Rasmussen, Ramona Trebbien

**Affiliations:** 1 Department of Infectious Disease Epidemiology and Prevention, Statens Serum Institut, Denmark; 2 National Influenza Centre for WHO, Department of Virus and Microbiological Special Diagnostics, Statens Serum Institut, Denmark; 3 Division of Infectious Disease Preparedness, Statens Serum Institut, Denmark

**Keywords:** Influenza surveillance, age distribution, influenza subtypes and lineages, infection incidence, hospitalisation incidence

## Abstract

**Background:**

Influenza was almost absent for 2 years following the implementation of strict public health measures to prevent the spread of SARS-CoV-2. The consequence of this on infections in different age groups is not yet known.

**Aim:**

To describe the age groups infected with the influenza virus in 2021/22, the first post-pandemic influenza season in Denmark, compared with the previous six seasons, and subtypes circulating therein.

**Methods:**

Infection and hospitalisation incidences per season and age group were estimated from data in Danish registries. Influenza virus subtypes and lineages were available from samples sent to the National Influenza Centre at Statens Serum Institut.

**Results:**

Test incidence followed a similar pattern in all seasons, being highest in 0–1-year-olds and individuals over 75 years, and lowest in 7–14-year-olds and young people 15 years to late twenties. When the influenza A virus subtypes A(H3N2) and A(H1N1)pdm09 co-circulated in seasons 2015/16 and 2017/18 to 2019/20, the proportion of A(H1N1)pdm09 was higher in 0–1-year-olds and lower in the over 85-year-olds  compared with the overall proportion of A(H1N1)pdm09 in these seasons. The proportion of A(H3N2) was higher in the over 85 years age group compared with the overall proportion of A(H3N2). The 2016/17 and 2021/22 seasons were dominated by A(H3N2) but differed in age-specific trends, with the over 85 years age group initiating the 2016/17 season, while the 2021/22 season was initiated by the 15–25-year-olds, followed by 7–14-year-olds.

**Conclusion:**

The 2021/22 influenza season had a different age distribution compared with pre-COVID-19 pandemic seasons.

**Key public health message**

**What did you want to address in this study and why?**

We wished to compare age groups infected with the influenza virus during the first post COVID-19 influenza season with the previous six seasons to understand how influenza A virus subtypes and influenza B virus lineages infected different age groups in different seasons. We also wanted to investigate the impact of influenza A subtypes and influenza B lineages on hospitalisation incidence in the different seasons.

**What have we learnt from this study?**

The different age groups were not equally infected with all influenza virus subtypes and lineages. In addition, the distribution of subtypes/lineages between age groups tended to change over time. Knowledge about subtypes and lineages dominating in previous seasons helps predict the upcoming season.

**What are the implications of your findings for public health?**

Based on these findings of influenza virus subtypes and lineages circulation in previous seasons and the age groups infected, we can predict who will likely be infected and hospitalised in upcoming seasons. This is valuable knowledge when planning resources for upcoming influenza seasons.

## Introduction

Influenza seasons in Denmark follow the usual pattern observed in temperate regions in the northern hemisphere [1]. The season often starts in December, peaks in January/February and gradually ends in March/April. In March 2020, strict public health measures were implemented in Denmark due to the COVID-19 pandemic, which abruptly ended the 2019/20 influenza season, and as a result, influenza was nearly absent with only sporadic cases detected [2]. At the end of January 2022, after nearly 2 years of various degrees of strict public health measures in Denmark, all restrictions were lifted and a surge in influenza cases occurred during the following weeks [3].

The strict public health measures implemented in most countries worldwide during the COVID-19 pandemic markedly impacted the occurrence of infectious diseases [4,5] and consequences for the population’s immunity still need to be fully elucidated. Changed seasonal patterns were observed for example for respiratory syncytial virus (RSV), where out-of-season epidemics after or in connection with the COVID-19 pandemic were reported in several countries worldwide [6-8]. A change in affected age groups was observed for RSV in some countries, which may be explained by a lack of build-up immunity in the younger age groups. The concept of immunity debt after less exposure to the usual infectious pathogens as a result of strict public health measures during the COVID-19 pandemic has previously been discussed [9]. It has been speculated how the lack of exposure to influenza could affect susceptibility to influenza virus infections in the population and whether age groups are affected differently [10].

Seasons where the influenza A(H3N2) virus dominates are often characterised by a higher rate of hospitalisation and mortality in the population above 65 years of age whereas seasons dominated by the influenza A(H1N1)pdm09 virus tend to affect the younger population to a higher degree [11-14]. There are two lineages of influenza virus type B, Yamagata and Victoria, however, since 2020, only B/Victoria has been observed worldwide. Since the 2019/20 influenza season, B/Victoria has mainly affected people below 50 years of age due to antigenic phenotype reversion [15].

Diagnostic test activity for influenza is usually highly variable among age groups. The youngest children and people aged 65 years and above have the highest test incidence in Denmark, whereas the test incidence in school children and adults below 65 years of age is lower [16]. The COVID-19 pandemic has led to concerns about changes in testing practices for influenza, and the potential implications for influenza surveillance at both national and global levels [17]. During the COVID-19 pandemic, different manufacturers of point of care testing (POCT) began to produce tests that detected both SARS-CoV-2 and influenza virus, providing the opportunity to ensure diagnostics for both pathogens on already established POCT platforms.

In this study we describe the 2021/22 influenza season, the first following the lifting of COVID-19 strict public health measures in Denmark, with focus on the age groups infected with the circulating virus strain compared with the previous six seasons (2015/16 to 2020/21). In addition, in all seven seasons we investigated which age groups were infected with the circulating influenza virus types and subtypes/lineages, age-specific test patterns and hospitalisation incidence.

## Methods

### Data sources

In Denmark, Statens Serum Institut (SSI) is responsible for conducting the national influenza surveillance. The surveillance system is important in alerting healthcare professionals when the influenza season starts and reports on epidemiological and laboratory findings throughout the season.

In the Danish Microbiology Database (MiBa), data on all patients tested for influenza A and B viruses by PCR, either by general practitioners (GPs) or at hospitals, are registered in real time [18]. Each sample is registered together with the unique personal identification number assigned to all individuals living in Denmark either at birth or at immigration [19]. The test result provides information on sampling date, whether the sample is positive or negative for influenza A virus and/or influenza B virus and the individual's age at the time of sampling. All clinical microbiological laboratories are instructed to submit a random subset of the influenza virus positive samples to the National Influenza Centre (NIC) at SSI for subtyping and genetic characterisation.

Information on hospital admission was available from the National Patient Registry (NPR) [20]. Linkage of data from NPR and MiBa allowed identification of patients who were admitted to hospital. Patients sampled within 4 days of, or during, an admission lasting 12 hours or more were considered hospitalised.

Age distribution (age in years) of the Danish population is available from Statistics Denmark (https://www.statistikbanken.dk/statbank5a/default.asp?w=2195) [21] and updated quarterly. This information was used to estimate influenza virus test incidence and influenza A virus and B virus infection incidence per 10,000 population by age in years per influenza season. In addition, population information was used to estimate influenza A virus and B virus infection and hospitalisation incidences per 10,000 population by age group and week of sampling within each influenza season.

### Data analysis

#### Data handling

An influenza season was defined as lasting from the start of week 40 in a given year to the end of week 39 in the following year. This study included seven influenza seasons from 2015/16 to 2021/22. All influenza virus test results during these seasons were extracted from MiBa.

Based on sampling date, positive and negative influenza virus samples were grouped by week, year and season of sampling. Age at sampling was grouped into 0–1, 2–6, 7–14, 15–44, 45–64, 65–74, 75–84 and over 85 years of age. For the 2021/22 season, the 15–44-year-old age group was further partitioned into 15–24 and 25–44 years of age due to a high influenza virus infection incidence in people younger than 20 years.

For the calculations of test incidence and influenza A and B virus infection incidence, only one test per week per person was included. A positive influenza A and/or influenza B virus PCR test result was prioritised over a negative test. Consecutive negative tests from one person from different weeks were included in the calculation of test incidence per week until an influenza virus positive test was detected. However, only the first positive swab for influenza A and/or B virus was included in the influenza virus incidence calculations. The calculation of influenza A and B virus hospitalisation incidence included a subset of influenza A and B virus infections where patients were hospitalised (defined as an admission lasting 12 hours or more).

All test incidences, influenza virus infection and hospitalisation incidences were calculated per 10,000 population using the whole Danish population as the denominator. Test incidences were calculated for all seven influenza seasons. Influenza virus infection and hospitalisation incidences were calculated for six seasons due to only 43 influenza A virus and 54 influenza B virus positive individuals detected during the 2020/21 season.

#### Subtyping

All samples received for further characterisation were subtyped A(H1N1)pdm09, A(H3N2) or B by an in-house designed multiplex RT-PCR assay targeting the matrix, haemagglutinin and neuraminidase segments. Quantification cycle (Cq) values below 40 were interpreted as positive. Some influenza A virus samples had Cq values above this threshold and were therefore not subtyped. A subset of influenza B virus positive samples were lineage determined (B/Victoria or B/Yamagata). Between 1,900 and 5,000 influenza virus positive samples per season were subtyped or lineage determined, which comprised 16%–40% of all influenza virus positive samples.

#### Presentation of data

Two sets of data were used for the analysis. The first dataset included all national influenza virus tests, both positive (for influenza virus A and/or B) and negative. Influenza virus test incidence and influenza A and B virus infection incidence per 10,000 population by age in years were calculated per season. In addition, positive influenza virus tests for the pre-defined age groups were presented as influenza A and B virus infection and hospitalisation incidences per week over time to explore the changes during the season in age groups infected and hospitalised with influenza. The second dataset was a subset of the national influenza A and B virus positive data, where subtypes and lineages were available to explore if the age groups were equally infected with the different subtypes and lineages and whether this changed over time. All data management were carried out in SAS version 9.4.

## Results

### Influenza testing during the 2015/16–2021/22 seasons

**Figure 1:**
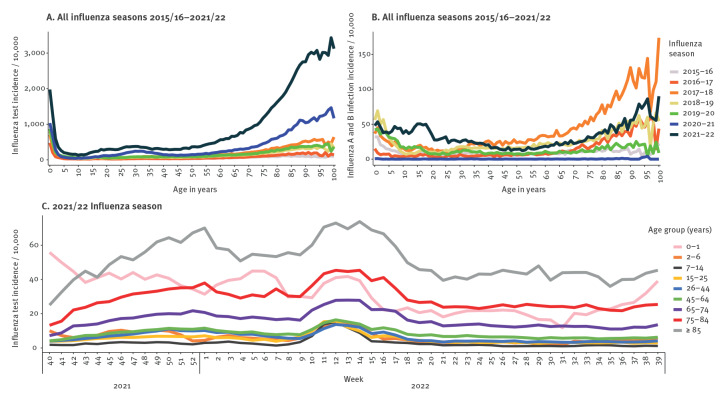
Influenza (A) test incidence by age in years, (B) infection incidence by age in years and (C) weekly test incidence per age group during the 2021/22 season, Denmark, influenza seasons 2015/16–2021/22

In Denmark, influenza testing increased from the 2015/16 season to the 2021/22 season. During all seasons, test incidence followed a similar pattern of highest test incidence in 0–1-year-olds and individuals over 75 years of age, and lowest test incidence in 7–14-year-old school children and in young people 15 years of age to late twenties (Figure 1A).

During the COVID-19 pandemic seasons 2020/21 and 2021/22, a distinct increase in influenza test incidence was observed, and during the 2021/22 season this increase was observed in all age groups. Despite a low overall influenza test incidence for 5–19-year-olds in the 2021/22 season, these ages had an influenza virus incidence similar to that observed for the ages 0 and 1-year-olds (Figure 1B). The weekly test incidence per age group during the 2021/22 season showed a parallel increase in test activity that was elevated from week 9 2021/22 to week 15 2021/22 for all age groups (Figure 1C).

In all seven influenza seasons, age distribution was similar in the national surveillance and in the subsample of samples submitted to SSI from regional laboratories for further characterisation.

### Influenza virus types and subtype/lineage distribution by age group during the 2015/16–2021/22 seasons

**Figure 2:**
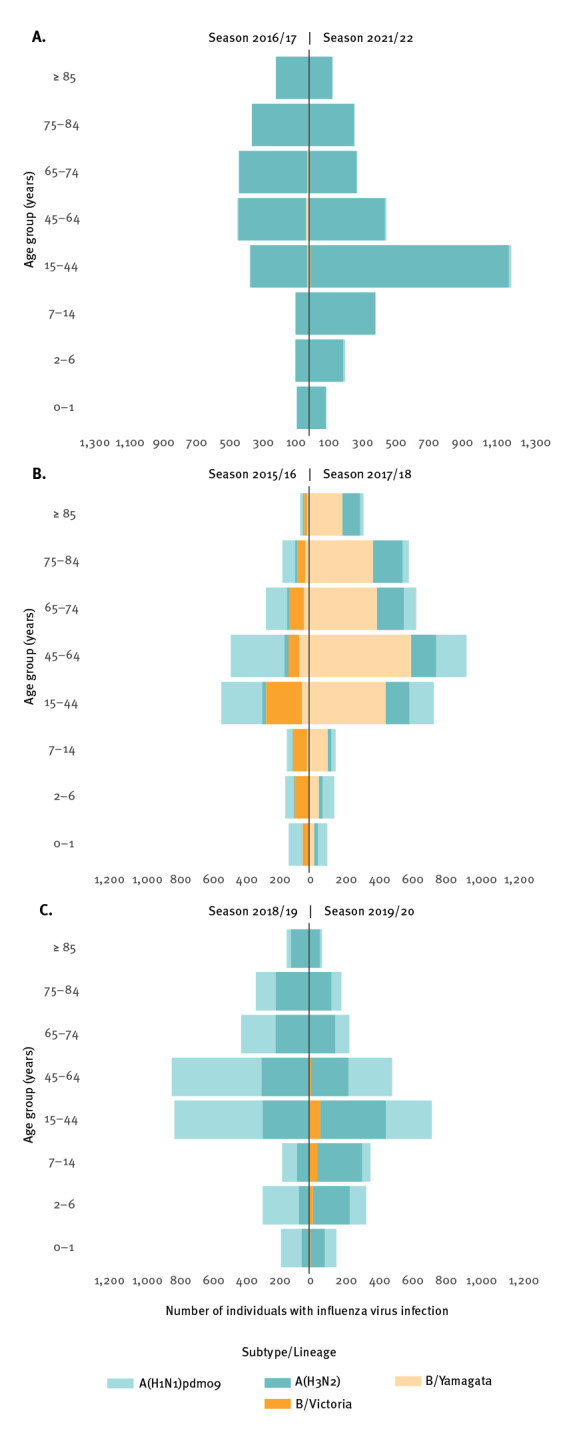
Number of individuals with influenza A(H3N2), A(H1N1)pdm09, B/Victoria and B/Yamagata infections by age group during (A) the 2016/17 and 2021/22 influenza seasons, (B) the 2015/16 and 2017/18 influenza seasons and (C) the 2018/19 and 2019/20 influenza seasons, Denmark

Influenza A(H3N2) dominated seasons, 2016/17 and 2021/22. The positive influenza virus samples subtyped at the NIC at SSI showed that the circulating subtype in the 2021/22 season was almost exclusively influenza A(H3N2) virus, which was also the case in the 2016/17 influenza season (Table 1). In the 2021/22 season, 62.3% (1,904/3,057) of all A(H3N2) samples further characterised at the NIC were obtained from age groups between 0 and 44 years-old. In the 2016/17 season, only 29.8% (579/1,942) of the characterised A(H3N2) samples originated from these age groups (Table 1, Figure 2A).

**Table 1:** Number of influenza A and B virus samples subtyped and lineage determined based on a random subsample from regional laboratories received at Statens Serum Institut, Denmark, influenza seasons 2016/17 and 2021/22

Age (years)	Influenza season 2016/17								Influenza season 2021/22							
	A(H1N1)pdm09		A(H3N2)		B/Victoria		B/Yamagata		A(H1N1)pdm09		A(H3N2)		B/Victoria		B/Yamagata	
	n	%^a^	n	%^a^	n	%^a^	n	%^a^	n	%^a^	n	%^a^	n	%^a^	n	%^a^
0–1	1	1.3	73	97.3	0	0.0	1	1.3	3	2.9	102	97.1	0	0.0	0	0.0
2–6	2	2.4	80	95.2	1	1.2	1	1.2	11	5.1	206	94.9	0	0.0	0	0.0
7–14	0	0.0	82	100.0	0	0.0	0	0.0	4	1.0	398	99.0	0	0.0	0	0.0
15–44	4	1.1	344	96.1	1	0.3	9	2.5	15	1.2	1,198	98.3	6	0.5	0	0.0
45–64	6	1.4	409	94.7	0	0.0	17	3.9	9	1.9	455	97.6	2	0.4	0	0.0
65–74	0	0.0	414	97.9	0	0.0	9	2.1	4	1.4	287	98.6	0	0.0	0	0.0
75–84	0	0.0	341	98.8	0	0.0	4	1.2	5	1.8	271	98.2	0	0.0	0	0.0
≥ 85	0	0.0	199	99.5	0	0.0	1	0.5	2	1.4	140	98.6	0	0.0	0	0.0
Total	13	0.7	1,942	97.2	2	0.1	42	2.1	53	1.7	3,057	98.0	8	0.3	0	0.0

^a^ Row % within season.

**Figure 3:**
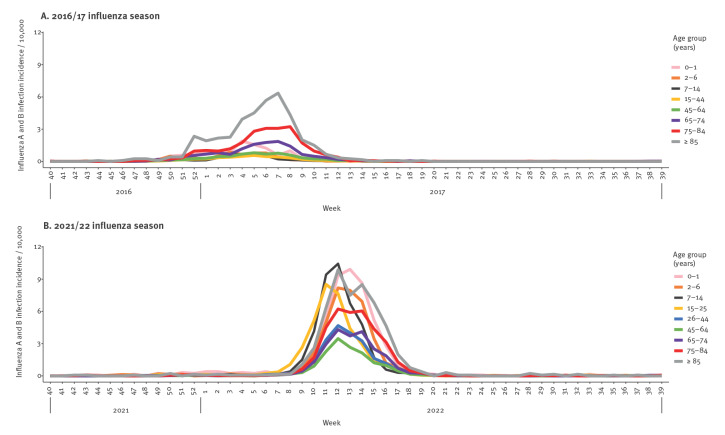
Combined influenza A and B virus infection incidence per 10,000 individuals per week and age group during (A) the 2016/17 influenza season and (B) the 2021/22 influenza season, Denmark

Comparison of weekly influenza virus infection incidence by age group during 2016/17 and 2021/22 showed marked difference in age distribution (Figure 3). In the 2016/17 season, the increase in influenza virus infections was first observed in the over 85-year-olds, who also had the highest influenza incidence overall, followed by the 75–84-year-olds, while influenza incidence in the other age groups remained at a lower level (Figure 3A). In the 2021/22 season, the increase in influenza virus infections was first observed in 15–25-year-olds (Figure 3B), and shortly after in 7–14-year-olds. This was then followed by an increase in influenza virus infections in those over 85 years and then an increase in remaining age groups.

### Influenza seasons with co-circulation of influenza A virus subtypes and influenza B virus lineages, 2015/16 and 2017/18–2019/20

Of the seven influenza seasons studied, four had co-circulation of influenza virus types and subtypes (Table 2). The 2015/16 season was dominated by influenza A(H1N1)pdm09 and influenza B/Victoria, with only a small proportion of A(H3N2) detected (Figure 2B). During the 2017/18 season, B/Yamagata was the dominant influenza virus type with co-circulations of A(H1N1)pdm09 and A(H3N2) (Figure 2B), while A(H1N1) pdm09 and A(H3N2) co-circulated in the following 2018/19 and 2019/20 seasons with only sporadic influenza B detected (Figure 2C). In all four seasons, the proportion of A(H1N1)pdm09 was higher in 0–1-year-olds and lower in the over 85 years age group compared with the overall proportion of A(H1N1) pdm09 in these seasons. At the same time, in all four seasons, the proportion of A(H3N2) was higher in the over 85 years age group compared with the overall proportion of influenza A(H3N2) (Table 2).

**Table 2:** Number of influenza A and B virus samples subtyped and lineage determined per age group in a random subsample received from regional laboratories at Statens Serum Institut, Denmark, influenza seasons 2015/16, 2017/18, 2018/19 and 2019/20

Age (years)	A(H1N1)pdm09		A(H3N2)		B/Victoria		B/Yamagata		A(H1N1)pdm09		A(H3N2)		B/Victoria		B/Yamagata	
	n	%^a^	n	%^a^	n	%^a^	n	%^a^	n	%^a^	n	%^a^	n	%^a^	n	%^a^
Influenza season 2015/16									Influenza season 2017/18							
0–1	83	67.5	2	1.6	36	29.3	2	1.6	56	50.9	24	21.8	0	0.0	30	27.3
2–6	52	36.1	6	4.2	83	57.6	3	2.1	71	46.7	23	15.1	0	0.0	58	38.2
7–14	32	23.9	5	3.7	85	63.4	12	9.0	27	16.9	21	13.1	0	0.0	112	70.0
15–44	247	46.7	23	4.4	217	41.0	42	7.9	147	19.6	143	19.0	2	0.3	460	61.2
45–64	324	68.6	24	5.1	64	13.6	60	1.7	183	19.3	151	15.9	1	0.1	614	64.7
65–74	127	48.9	16	6.2	88	33.9	29	11.2	75	11.6	163	25.2	0	0.0	408	63.2
75–84	77	47.8	14	8.7	47	29.2	23	14.3	37	6.2	179	29.8	1	0.2	384	63.9
≥ 85	17	31.5	8	14.8	19	35.2	10	18.5	20	6.1	107	32.6	0	0.0	201	61.3
Total	959	51.1	98	5.2	639	34.0	181	9.6	616	16.7	811	21.9	4	0.1	2,267	61.3
Influenza season 2018/19									Influenza season 2019/20							
0–1	125	73.5	43	25.3	1	0.6	1	0.6	70	42.4	87	52.7	8	4.9	0	0.0
2–6	218	77.9	62	22.1	0	0.0	0	0.0	100	29.0	222	64.4	23	6.7	0	0.0
7–14	91	55.8	70	42.9	2	1.2	0	0.0	50	13.5	271	73.2	49	13.2	0	0.0
15–44	532	65.5	277	34.1	3	0.4	0	0.0	278	37.6	394	53.2	66	8.9	2	0.3
45–64	541	65.3	286	34.5	1	0.1	0	0.0	264	52.7	225	44.9	12	2.4	0	0.0
65–74	208	50.7	201	49.0	0	0.0	1	0.2	86	35.4	154	63.4	3	1.2	0	0.0
75–84	121	37.7	200	62.3	0	0.0	0	0.0	60	30.8	134	68.7	1	0.5	0	0.0
≥ 85	25	18.5	109	80.7	1	0.7	0	0.0	12	15.4	66	84.6	0	0.0	0	0.0
Total	1,861	59.7	1,248	40.0	8	0.3	2	0.1	920	34.9	1,553	58.9	162	6.1	2	0.1

^a^ Row % within season.

**Figure 4:**
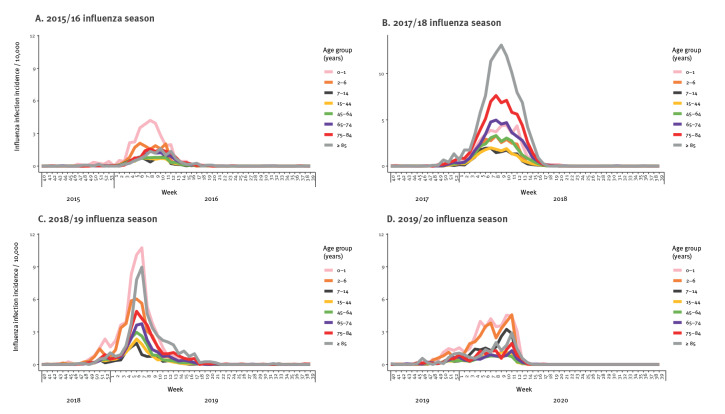
Combined influenza A and B virus infection incidence per 10,000 per week and age group during (A) the 2015/16 influenza season (B) the 2017/18 influenza season, (C) the 2018/19 influenza season and (D) the 2019/20 influenza season, Denmark

These differences in influenza A virus subtype distributions between age groups also appear in Figure 3. In seasons 2015/16 and 2018/19, dominated by A(H1N1) pdm09, 0–1-year-old children had the highest influenza virus infection incidence, 30.64 and 52.77 per 10,000 individuals, respectively (see Supplementary Table S1). In season 2018/19, A(H3N2) was also circulating which resulted in an influenza incidence of 48.00 per 10,000 individuals among the over 85-year-olds (Supplementary Table S1). In the 2019/20 season, A(H1N1)pdm09 circulated during the beginning of the season resulting in an initial increase in incidence among the 0–1 and 2–6-year-olds which was followed by circulation of A(H3N2) and an increase in incidence in 7–14 and over 85-year-olds (Figure 4). Similar to the 2021/22 season, an age shift in A(H3N2) infections was also observed during the 2019/20 season where 62.7% (974/1,553) of all A(H3N2) samples originated from the age groups between 0 and 44 years (Figure 2C). Influenza B/Victoria was circulating in the 2015/16 season where this B-lineage was detected in all age groups (Figure 2A). In contrast, during the 2019/20 season B/Victoria mainly affected the groups below 45 years of age (Table 2) (Figure 2C). The influenza B/Yamagata only dominated in the 2017/18 season, which did not allow indication of an age trend.

Overall, influenza seasons were initiated either by the 0–1 and/or the 2–6-year-old age groups in seasons 2015/16, 2018/19 and 2019/20, or by the over 85 years age group in seasons 2016/17 and 2017/18. Only in the 2021/22 season was the season initiated by young adults (15–25-year-olds) and school children (7–14-year-olds).

### Hospitalisation per age group during the 2015/16­–2019/20 and 2021/22 influenza seasons

**Figure 5:**
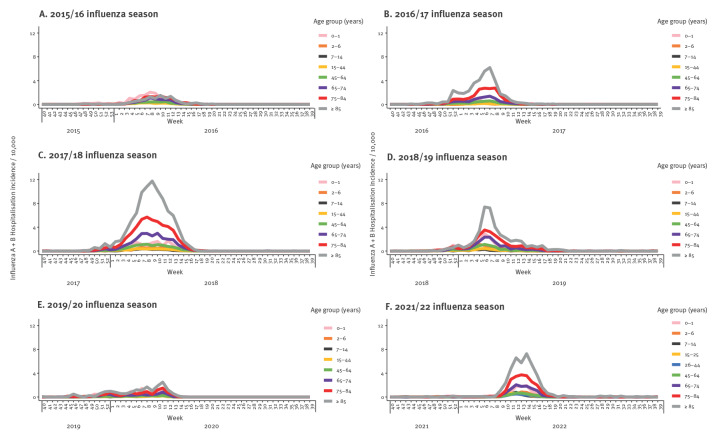
Combined influenza A virus and influenza B virus hospitalisation incidence per 10,000 individuals per week and age group during (A) the 2015/16 influenza season, (B) the 2016/17 influenza season, (C) the 2017/18 influenza season, (D) the 2018/19 influenza season, (E) the 2019/20 influenza season and (F) the 2021/22 influenza season, Denmark

Overall, influenza hospitalisation incidence varied between age groups and between seasons, with the highest incidence observed among the over 85-year-olds (10.6–91.3/10,000) and 75–84-year-olds (10.9–49.7/10,000). The highest hospitalisation incidence was observed in the 2017/18 season dominated by B/Yamagata (Supplementary Table S1 and Figure 5C). In seasons dominated by A(H3N2) (2021/22 and 2016/17) and in the season 2018/19 with co-circulation of A(H1N1) and A(H3N2), similar hospitalisation incidences were observed in the over 85 and 75–84-year-olds ranging from 38.3 to 41.5 per 10,000 and 20.2 to 24.9 per 10,000, respectively (Supplementary Table S1 and Figure 5BDF). In the 2018/19 and 2019/20 seasons, A(H3N2) accounted for 40% and 60%, respectively, of all influenza detected (Table 2). These two seasons were similar with respect to test activity. However, the number of individuals testing positive for either influenza A or B virus was higher in the 2018/19 season, leading to infection and hospitalisation incidences in the over 85 years age group of 48.0 and 41.5 per 10,000 (Supplementary Table S1) compared with infection and hospitalisation incidences of 19.4 and 17.6 per 10,000 in the over 85 years age group in the 2019/20 season (Supplementary Table S1).

In younger individuals (2–44 years), hospitalisation incidence was similar across seasons and varied between 0.7 and 6.8 per 10,000. In 0–1-years-olds, hospitalisation incidence varied from 10.1 to 16.8 per 10,000 and was highest in the seasons where A(H1N1)pdm09 was the most prevalent influenza A subtype (2015/16 and 2018/19) and where B/Yamagata was the dominant influenza B lineage (Supplementary Table S1).

## Discussion

Following the lifting of the COVID-19 restrictions, the 15–25-year-olds followed by the 7–14-year-olds initiated the increase in influenza in Denmark during the atypically late 2021/22 season starting in February 2022. This was unusual compared with previous seasons where either the youngest age groups (0–1 or 2–6-year-olds) or the over 85 years age group were initially affected at the start of the seasons. The steep increase in influenza virus infection incidence in 2021/22 among the 15–25 and 7–14-year-olds was not due to an isolated increase in test incidence in these age groups. A simultaneous increase in test incidence was observed in all age groups during the 2021/22 season with an overall lower test incidence among children and young adults.

It could be speculated that the shift in initially affected age groups might be attributed to the lack of built-up immunity in these age groups after a 2-year absence of seasonal influenza, similar to the age shift observed for RSV in the 2021/22 RSV season [22]. However, the majority of children and young adults in these two groups were likely exposed to A(H3N2) during the 2016/17 season. In addition, A(H3N2) was also circulating during the 2019/20 season where more than 50% of the influenza virus detected in the 7–14 and 15–44-year-olds was A(H3N2).

On 15 December 2021, schools closed in Denmark and all schoolchildren in primary and lower secondary schools began online learning. On 5 January 2022, children and adolescents were allowed to return to full in-person learning, with the condition that pupils and staff be tested for SARS-CoV-2 twice a week. However, on 1 February 2022, all restrictions were lifted [23]. It is likely that this may have resulted in more mixing and interaction in the schools. Furthermore, there were no longer restrictions to stay home in case of mild respiratory symptoms, which also may have contributed to increased transmission of the influenza virus among children and adolescents, followed by the spread of influenza virus to other age groups. From a virological point of view, antigenic drift of the A(H3N2) viruses may also be a contributing factor, as distinct antigenic/genetic clades were in circulation in seasons prior to the 2021/22 season [24].

We also observed that when the influenza A virus subtypes A(H3N2) and A(H1N1)pdm09 co-circulated, the proportion of A(H1N1)pdm09 was higher in 0–1-year-olds and lower in the over 85 years age group compared with the overall proportion of A(H1N1)pdm09. This may be explained by prior exposure to the influenza A(H1N1)pdm09 pandemic strain among the older age groups [25]. An overall higher proportion of A(H3N2) in the age groups over 65 years was also reported in a study from Singapore [26]. Influenza seasons dominated by A(H3N2) are among the seasons with the highest influenza hospitalisation incidence, with the majority of hospitalisations in those over 75 years of age. Seasons 2018/19 and 2019/20 were comparable in relation to test activity, and in both seasons influenza A(H3N2) and A(H1N1)pdm09 co-circulated. In the 2018/19 season, 64% (796/1,248) of the A(H3N2) cases were detected in the age groups above 45 years of age, however, during the 2019/20 season, 63% (774/1,553) of A(H3N2) cases were detected in the 0–44 years age group. This shift in age may have been one of the reasons why the hospitalisation incidence was much lower during the 2019/20 season compared with the 2018/19 season. This age shift in A(H3N2) is similar to the age shift observed for A(H3N2) during the 2021/22 season, where 63% (1,823/2,874) of A(H3N2) cases were detected in individuals aged between 0 and 44 years. The reason for this age shift during the past two influenza seasons is not known. However, the immunological imprinting in the different age groups for A(H3N2) may be part of the explanation [27]. An age shift was also observed for B/Victoria, from infection in all age groups in the 2015/16 season to infection mainly in those below 44 years of age in 2019/20. This was explained by antigenic phenotype reversion [15].

Influenza virus test incidence varied between the seasons included in this study. Influenza virus positivity incidence is highly correlated with test incidence and therefore it is not possible to directly compare the magnitude of influenza virus infection incidence from year to year. However, as the test pattern across age groups was very similar between seasons, the distribution of influenza virus detections between age groups can be compared between seasons. Therefore, it is not likely that differences in age distributions between seasons are due to differences in test patterns. However, it could be speculated that if people in the age groups 7–14 and 15–25-years were generally tested more, the actual transmission of influenza in this population may differ from the observed transmission, and therefore, some seasons may have been initiated by these age groups. This is important from a public health perspective when preparing interventions to prevent the spread of influenza. In addition, individuals referred to hospitals with influenza-like symptoms will, according to Danish national guidelines, be tested for influenza, which makes the magnitude of influenza virus hospitalisation incidence more comparable between seasons. Percentage of influenza virus positive tests is another measure of exploring differences between age groups. However, this measure is often inverse correlated with test activity, therefore incidences were used instead of percentage positive.

In the 2021/22 season, the children in the 2–6 years age group were vaccinated for the first time. It could be speculated that this may have changed transmission in the younger age groups. Although influenza vaccine effectiveness in 2–6-year-olds was 62.4 (95% confidence interval (CI): 50.5–74.1) in non-hospitalised children [28], vaccine coverage in this age group was only 29% [29] and an increase in influenza cases in 2–6-year-olds were observed shortly after the increase in 7–14-year-olds. Furthermore, the 2–6-year-olds were not the age group that initiated the transmission of A(H3N2) in previous seasons. It seems likely that people in the age group of 15–25 years were the primary source of influenza virus transmission to other age groups.

In all the seasons where influenza A(H3N2) virus was circulating either as the dominant subtype or co-circulating with A(H1N1)pdm09 virus, the older age groups (75–84 years and over 85 years) had the highest hospitalisation incidence. Although we compared the 2018/19 and 2019/20 seasons where A(H3N2) and A(H1N1)pdm09 co-circulated, infection and hospitalisation incidences varied between these seasons indicating that factors other than the circulating stains could play a role. Influenza vaccine effectiveness (VE) cannot explain these observed differences as VE estimates from the 2018/19 and 2019/20 seasons were estimated to be low against A(H3N2) and moderate against A(H1N1)pdm09 [30,31].

This study has some limitations. The surveillance of influenza in Denmark is a national surveillance where there are only minor changes in the population during a season. However, for this study, no other variables than those reported were available to indicate whether the health of those infected with influenza within an age group differed between the start and end of a season. A strength of this study is the comparison of the age distribution of influenza virus infection incidence during the first post pandemic season dominated by A(H3N2) with another A(H3N2)-dominated season 2016/17, and the comparison of age distributions between seasons with various subtypes/lineages co-circulating.

## Conclusion

Knowledge about which influenza virus types and subtypes/lineages that circulated in previous seasons and the age groups infected can help predict which influenza viruses will likely be circulating in the upcoming season and thereby which age groups may be affected. This is valuable information when planning hospital capacity and other resources for upcoming influenza seasons. A large proportion of A(H3N2) and B/Victoria virus infections in the younger age groups in 2019/20 indicates that the distribution of virus subtypes/lineages between age groups might not be as fixed as expected. It is also important to be aware of possibly undertested age groups that may play an undetected role in influenza transmission.

The COVID-19 pandemic greatly impacted the circulation of usual infectious diseases, since strict public health measures put in place during the pandemic were highly effective in preventing influenza in Denmark and many other countries. In this study, we observed that the late influenza season in 2021/22 in Denmark exhibited a different age distribution pattern compared with six previous pre-COVID-19 seasons, even though the diagnostic test pattern was similar.

## Supplementary Data

124000 Supplementary Material
