# Supplementary material for: Age-dependent influenza infection patterns and subtype circulation in Denmark, in seasons 2015/16 to 2021/22
Source: Euro Surveill. 2024 Jan 25;29(4):2300263. doi: 10.2807/1560-7917.ES.2024.29.4.2300263 (PMC10986648; doi:10.2807/1560-7917.ES.2024.29.4.2300263)
Supplement: Supplementary Material [file 23-00263_EMBORG_Supplementary_material.pdf]

This supplementary material is hosted by *Eurosurveillance* as supporting information alongside the article ’Age-dependent influenza infection patterns and subtype circulation in Denmark, in seasons 2015/16 to 2021/22’, on behalf of the authors, who remain responsible for the accuracy and appropriateness of the content. The same standards for ethics, copyright, attributions and permissions as for the article apply. Supplements are not edited by *Eurosurveillance* and the journal is not responsible for the maintenance of any links or email addresses provided therein.

Table S1. Influenza test and infection incidences per age group in the seasons 2015/16 to 2021/22

| | 2021/22 | | | | | | 2020/21 | | | | | | 2019/20 | | | | | |
|---|---|---|---|---|---|---|---|---|---|---|---|---|---|---|---|---|---|---|
| | | | | Incidence/10,000 | | | | | | Incidence/10,000 | | | | | | Incidence/10,000 | | |
| | Total tested | InfluA | InfluB | Test | A+B positive | A+B hosp | Total tested | InfluA | InfluB | Test | A+B positive | A+B hosp* | Total tested | InfluA | InfluB | Test | A+B positive | A+B hosp |
| 0-1 | 17644 | 621 | 8 | 1410.21 | 50.27 | 10.55 | 10461 | 4 | 6 | 851.50 | 0.81 | - | 8279 | 479 | 50 | 671.01 | 42.87 | 10.05 |
| 2-6 | 8677 | 1206 | 22 | 280.78 | 39.74 | 4.34 | 4017 | 0 | 2 | 131.36 | 0.07 | - | 3798 | 904 | 153 | 125.80 | 35.01 | 3.61 |
| 7-14 | 7416 | 2126 | 10 | 144.93 | 41.74 | 2.78 | 2610 | 2 | 1 | 50.18 | 0.06 | - | 3022 | 828 | 195 | 57.25 | 19.38 | 1.69 |
| 15-44 | 61643 | 6095 | 36 | 282.05 | 28.05 | 2.92 | 35497 | 18 | 14 | 163.53 | 0.15 | - | 16290 | 1860 | 292 | 74.83 | 9.89 | 1.46 |
| 45-64 | 59139 | 2398 | 14 | 382.33 | 15.59 | 3.64 | 27430 | 10 | 10 | 177.62 | 0.13 | - | 16103 | 1217 | 54 | 104.79 | 8.27 | 2.37 |
| 65-74 | 46313 | 1469 | 4 | 736.04 | 23.41 | 11.59 | 21151 | 3 | 7 | 331.92 | 0.16 | - | 10399 | 649 | 26 | 161.52 | 10.48 | 5.92 |
| 75-84 | 56291 | 1561 | 8 | 1293.01 | 36.04 | 21.98 | 24035 | 3 | 8 | 583.89 | 0.27 | - | 9733 | 546 | 11 | 250.98 | 14.36 | 10.60 |
| +85 | 29388 | 691 | 1 | 2249.39 | 52.97 | 41.41 | 13165 | 3 | 6 | 1033.61 | 0.71 | - | 4558 | 234 | 7 | 366.54 | 19.38 | 17.61 |
| Total | 286511 | 16167 | 103 | 487.81 | 27.70 | 6.53 | 138366 | 43 | 54 | 236.93 | 0.17 | - | 72182 | 6717 | 788 | 123.97 | 12.89 | 3.46 |

| | 2018/19 | | | | | | 2017/18 | | | | | | 2016/17 | | | | | | 2015/16 | | | | | |
|---|---|---|---|---|---|---|---|---|---|---|---|---|---|---|---|---|---|---|---|---|---|---|---|---|
| | | | | Incidence/10,000 | | | | | | Incidence/10,000 | | | | | | Incidence/10,000 | | | | | | Incidence/10,000 | | |
| | Total tested | InfluA | InfluB | Test | A+B positive | A+B hosp | Total tested | InfluA | InfluB | Test | A+B positive | A+B hosp | Total tested | InfluA | InfluB | Test | A+B positive | A+B hosp | Total tested | InfluA | InfluB | Test | A+B positive | A+B hosp |
| 0-1 | 7970 | 773 | 4 | 643.89 | 62.77 | 16.88 | 6537 | 290 | 217 | 526.16 | 40.81 | 14.33 | 4107 | 136 | 8 | 338.64 | 11.87 | 4.95 | 3898 | 198 | 159 | 334.60 | 30.64 | 13.91 |
| 2-6 | 3658 | 1212 | 8 | 121.86 | 40.64 | 6.83 | 2859 | 369 | 422 | 95.70 | 26.48 | 5.05 | 1513 | 176 | 10 | 49.72 | 6.11 | 1.15 | 1889 | 173 | 307 | 61.13 | 15.53 | 3.75 |
| 7-14 | 2149 | 594 | 9 | 40.28 | 11.30 | 1.61 | 2367 | 197 | 665 | 44.03 | 16.03 | 2.60 | 1212 | 211 | 17 | 22.61 | 4.25 | 0.71 | 1356 | 80 | 276 | 25.36 | 6.66 | 1.42 |
| 15-44 | 13953 | 2975 | 47 | 63.95 | 13.85 | 2.81 | 13898 | 1123 | 2633 | 63.77 | 17.24 | 3.85 | 6709 | 921 | 69 | 30.82 | 4.55 | 1.35 | 6202 | 611 | 824 | 28.62 | 6.62 | 2.34 |
| 45-64 | 13796 | 2791 | 23 | 90.14 | 18.39 | 6.89 | 14673 | 1097 | 3153 | 96.20 | 27.86 | 10.49 | 6921 | 943 | 92 | 45.67 | 6.83 | 3.21 | 5897 | 702 | 397 | 39.16 | 7.30 | 3.89 |
| 65-74 | 9459 | 1456 | 22 | 145.85 | 22.79 | 14.82 | 10902 | 679 | 2054 | 168.31 | 42.19 | 25.71 | 5191 | 832 | 46 | 80.53 | 13.62 | 9.74 | 4245 | 315 | 331 | 66.66 | 10.14 | 7.46 |
| 75-84 | 8010 | 1146 | 15 | 218.92 | 31.73 | 24.87 | 9286 | 590 | 1686 | 267.17 | 65.48 | 49.69 | 4124 | 747 | 17 | 124.59 | 23.08 | 20.15 | 2868 | 178 | 218 | 89.84 | 12.40 | 10.90 |

| | | | | | | | | | | | | | | | | | | | | | | | | |
|---|---|---|---|---|---|---|---|---|---|---|---|---|---|---|---|---|---|---|---|---|---|---|---|---|
| +85 | 3792 | 579 | 5 | 311.69 | 48.00 | 41.51 | 4814 | 334 | 935 | 398.62 | 105.08 | 91.83 | 2034 | 472 | 10 | 170.08 | 40.30 | 38.30 | 1083 | 58 | 73 | 91.48 | 11.07 | 10.56 |
| Total | 62787 | 11526 | 133 | 108.14 | 20.08 | 7.83 | 65336 | 4679 | 11765 | 113.01 | 28.44 | 12.82 | 31811 | 4438 | 269 | 55.34 | 8.19 | 4.64 | 27438 | 2315 | 2585 | 48.08 | 8.59 | 4.19 |
